# Anti-Mycobacterial Nucleoside Antibiotics from a Marine-Derived *Streptomyces* sp. TPU1236A

**DOI:** 10.3390/md12126102

**Published:** 2014-12-17

**Authors:** Ying-Yue Bu, Hiroyuki Yamazaki, Kazuyo Ukai, Michio Namikoshi

**Affiliations:** Faculty of Pharmaceutical Sciences, Tohoku Pharmaceutical University, Aoba-ku, Sendai 981-8558, Japan; E-Mails: 21252502@is.tohoku-pharm.ac.jp (Y.-Y.B.); ukai_k@tohoku-pharm.ac.jp (K.U.); mnami@tohoku-pharm.ac.jp (M.N.)

**Keywords:** marine-derived actinomycete, *Streptomyces* sp., streptcytosines A–E, anti-mycobacterium activity, amicetin

## Abstract

Five new nucleoside antibiotics, named streptcytosines A–E (**1**–**5**), and six known compounds, de-amosaminyl-cytosamine (**6**), plicacetin (**7**), bamicetin (**8**), amicetin (**9**), collismycin B (**10**), and SF2738 C (**11**), were isolated from a culture broth of *Streptomyces* sp. TPU1236A collected in Okinawa, Japan. The structures of new compounds were elucidated on the basis of their spectroscopic data (HRFABMS, IR, UV, and 2D NMR experiments including ^1^H-^1^H COSY, HMQC, HMBC, and NOESY spectra). Streptcytosine A (**1**) belonged to the amicetin group antibiotics, and streptcytosines B–E (**2**–**5**) were derivatives of de-amosaminyl-cytosamine (**6**), 2,3,6-trideoxyglucopyranosyl cytosine. Compound **1** inhibited the growth of *Mycobacterium smegmatis* (MIC = 32 µg/mL), while compounds **2**–**5** were not active at 50 µg/disc. Bamicetin (**8**) and amicetin (**9**) showed the MICs of 16 and 8 µg/mL, respectively.

## 1. Introduction

Tuberculosis (TB) caused by *Mycobacterium tuberculosis* is still one of the main infectious diseases all over the world including with the human immunodeficiency virus (HIV) and malaria [1,2,3]. It was estimated that there were about 9 million clinical cases, 1.3 million deaths from TB, and 0.3 million deaths from HIV-co-infected TB in 2012 [4]. The development of multidrug-resistant TB (MDR-TB) has been observed as resistance against potent first-line drugs (rifampicin and isoniazid), and 450,000 patients were revealed to be infected by MDR-TB in 2012. Moreover, approximately 9.6% of these drug-resistant strains of *T. tuberculosis* were extensive drug-resistant TB (XDR-TB), which additionally shows resistance to some second-line drugs such as fluoroquinones, amikacin, kanamycin, and capreomycin. More recently, totally drug resistant TB (TDR-TB), which is resistant to all second-line drugs, has often been reported in India and other Asian countries [1,2,3,4,5]. Although a new drug, delmanid (Deltyba^®^), which inhibits the cell wall biosynthesis of mycobacteria, was approved in 2014 for the treatment of MDR-TB in EU and Japan [6,7], continuous efforts to discover new anti-TB agents with novel mechanisms of action and structural features are the emergent global demand.

**Figure 1:**
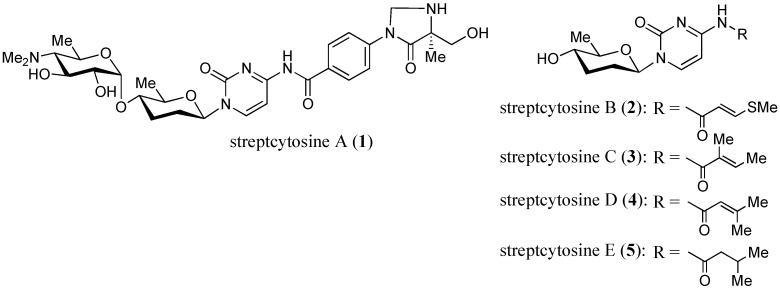
Structures of new compounds **1**–**5** isolated from *Streptomyces* sp. TPU1236A.

**Figure 2:**
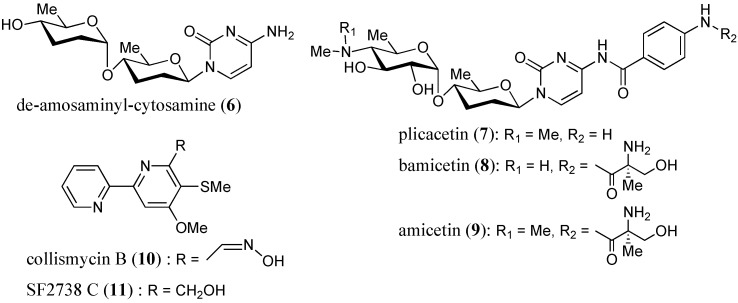
Structures of known compounds **6**–**11** isolated from *Streptomyces* sp. TPU1236A.

In the course of our studies on anti-TB metabolites from marine invertebrates and microorganisms, we have tested the culture broths of 50 marine-derived actinomycetes against *Mycobacterium smegmatis* NBRC 3207 and found that *Streptomyces* sp. strain TPU 1236A exhibited prominent activity. *M. smegmatis* has been used most widely in the search for anti-mycobacterial substances due to its fast-growing and non-pathogenic properties [8]. Bioassay-guided isolation from the culture broth of strain TPU1236A yielded five new compounds, designated as streptcytosines A–E (**1**–**5**) (Figure 1), together with six known compounds, de-amosaminyl-cytosamine (**6**) [9], plicacetin (**7**) [10], bamicetin (**8**) [11,12,13], amicetin (**9**) [9,11,12,13], collismycin B (**10**) [14,15], and SF2738 C (**11**) [15] (Figure 2). The isolation and anti-mycobacterial activities of compounds **1**–**11** have been described in this study.

## 2. Results and Discussion

The strain TPU1236A was isolated from a seawater sample collected at Iriomote Island in Okinawa, Japan. The partial sequence of the 16S rRNA gene used for identification showed 100% identity with those of *Streptomyces badius* and *Streptomyces sindenensis*. The bacterium was cultured by shaking in a liquid medium for 7 days at 25 °C in the dark.

The extracts of broth filtrates and mycelia were subjected to ODS silica gel column chromatography followed by preparative HPLC separation, as described in the Experimental Section, to afford compounds **1**–**11** (Figure 1 and Figure 2). Six known compounds were identified by their structures by comparing the spectroscopic data for **6**–**11** with those of the reported values for de-amosaminyl-cytosamine (**6**), plicacetin (**7**), bamicetin (**8**), amicetin (**9**), collismycin B (**10**), and SF2738 C (**11**), respectively [9,10,11,12,13,14,15,16,17].

### 2.1. Structure of Streptcytosine A (**1**)

**Figure 3:**
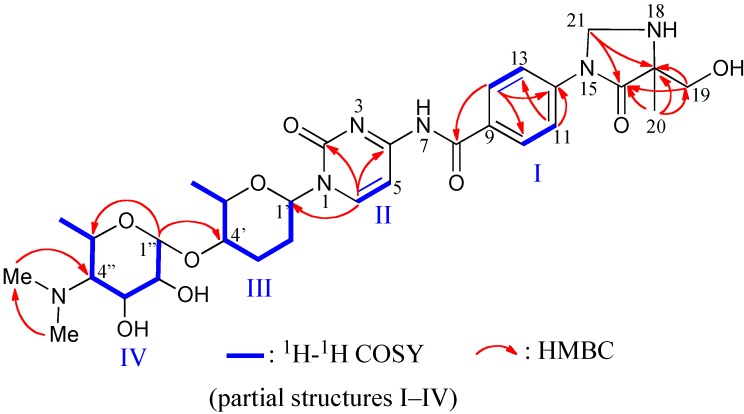
^1^H-^1^H COSY and key HMBC correlations for compound **1**.

Compound **1** was obtained as a colorless oil. Its molecular weight and formula (630, C_30_H_4__2_N_6_O_9_) were determined from HRFABMS and NMR data. ^1^H and ^13^C NMR signals were assigned by an analysis of ^1^H-^1^H COSY, HMQC, and HMBC data (Table 1). Partial structures I–IV were established from ^1^H-^1^H COSY data, and connectivity between these partial structures were elucidated by the HMBC correlations (Figure 3).

The chemical shifts and couplings of two sugar moieties (amosamine and amicetose units) and two aromatic rings (cytosine and PABA units) were very similar to those of amicetin (**9**) [9]. Differences in the molecular formulas and weights of **1** and **9** were only one carbon atom and 12 Da, which was detected at δ 61.1 in the ^13^C NMR spectrum of **1**. The protons attached to this carbon were observed as a broad singlet (2H) at δ 5.28, which showed HMBC correlations to C-16 and 17 (Figure 3). HMBC correlations from H_3_-19 to C-16, 17, and 20 and from H_2_-20 to C-16 and 17 along with the above data allowed to assign the partial structure of 4-imidazolidinone. Consequently, the skeletal structure of compound **1** was assigned as Figure 3 and named streptcytosine A.

**Table 1:** ^13^C (100 MHz) and ^1^H (400 MHz) NMR data for streptcytosine A (**1**) (CD_3_OD).

C#	δ_C_	δ_H_*,* mult. (*J* in Hz)	HMBC
2	157.4		
4	165.0		
5	99.1	7.58, d, (7.5)	6
6	146.7	8.21, d, (7.5)	2, 4, 5, 1′
8	168.4		
9	131.6		
10	130.8	8.10, d, (8.8)	8, 11, 12, 14
11	120.6	7.87, d, (8.8)	10, 13
12	142.0		
13	120.6	7.87, d, (8.8)	11, 14
14	130.8	8.10, d, (8.8)	8, 10, 12, 13
16	172.2		
17	68.3		
19	66.1	(a) 3.83, d, (11.6)	16, 17
		(b) 4.03. d, (11.6)	16
20	17.6	1.57, s	16, 17, 19
21	61.1	5.28, br s	16, 17
1′	84.9	5.79, d, (7.8)	
2′	31.1	(a) 2.19, br d, (9.0)	4′
		(b) 1.71, m	4′
3′	28.1	(a) 1.71, m	1′
		(b) 2.41, m	
4′	76.7	3.46, m	
5′	78.4	3.77, dq, (9.0, 6.0)	4′
6′	19.3	1.39, d, (6.0)	4′, 5′
1″	96.8	5.03, d, (3.6)	4′, 3″, 5″
2″	74.0	3.56, dd, (9.1, 3.6)	3″
3″	68.1	3.98, dd, (11.0, 9.1)	2″, 4″
4″	72.1	3.12, dd (11.0, 10.0)	3″, 5″, 6″, 7″, 8″
5″	64.2	4.10, dq, (10.0, 6.2)	
6″	19.1	1.47, d, (6.2)	4″, 5″
7″	42.6	3.01, s	4″, 8″
8″	42.6	3.01, s	4″, 7″

Since streptcytosine A (**1**) was obtained together with plicacetin (**7**), bamicetin (**8**), and amicetin (**9**) from the strain TPU1236A, the absolute configuration of the sugar moiety will be the same. The configuration of **1** at the C-17 position was also assigned to be the same as those of **8** and **9**, since these compounds should be biosynthesized by the same pathway. Moreover, compounds **1** (+72), **7** (+96), **8** (+66), and **9** (+76) showed the same positive specific rotation. Therefore, streptcytosine A (**1**) may share the same configuration for whole asymmetric carbons as compounds **8** and **9**, as depicted in Figure 1 and Figure 2.

### 2.2. Structures of Streptcytosines B–E (**2**–**5**)

**Figure 4:**
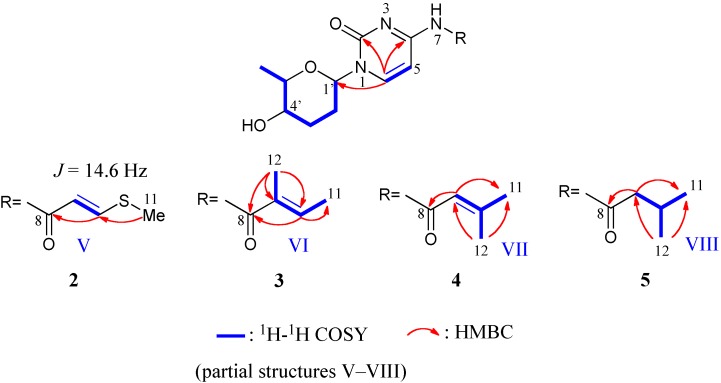
^1^H-^1^H COSY and key HMBC correlations for compounds **2**–**5**.

The molecular formulas and weights of compounds **2** (C_14_H_19_N_3_O_4_S, 325), **3** (C_15_H_21_N_3_O_4_, 307), **4** (C_15_H_21_N_3_O_4_, 307), and **5** (C_15_H_2__3_N_3_O_4_, 309) were determined from the HRFABMS and NMR data (Table 2). The presence of amicetose and cytosine moieties in **2**–**5** was assigned by the analysis of ^1^H-^1^H COSY and HMBC spectra (Figure 4). The ^1^H and ^13^C NMR signals due to two moieties in **2**–**5** were very similar to those in de-amosaminyl-cytosamine (**6**) [9]. Therefore, compounds **2**–**5** had the same amicetosylcytosine unit, and differences were observed in the amide moieties.

Streptcytosine B (**2**) had an *S*-Me group and 2-butenoyl unit. HMBC correlations from H_3_-11 to C-10, H-10 to C-8, and from H-9 to C-8 revealed the partial structure V as the amide moiety in **2**. The orientation of the double bond was assigned as *E* from the coupling constant (14.6 Hz) between H-9 and 10.

The 2-methyl-2-butenoyl unit (partial structure VI) in streptcytosine C (**3**) was elucidated from ^1^H-^1^H COSY and HMBC data (Figure 4). An NOE correlation was observed between H-10 and H_3_-11, but no cross peak was detected between H-10 and H_3_-12. These observations on NOEs were the same as cytosaminomycin D, which has a tiglic acid (2-methyl-2*E*-butenoic acid) moiety attached to the PABA unit by an amide bond [18]. Moreover, chemical shifts due to C-11 (δ 14.7) and 12 (12.2) of **3** resembled those of cytosaminomycin D (14.5 and 12.2), which was compared the chemical shifts with those of tiglic acid (13.83 and 10.95) and angelic acid (15.93 and 20.22, 2-methyl-2*Z*-butenoic acid). Thus, the orientation of the double bond was assigned as *E*.

Streptcytosine D (**4**) had a 3-methyl-2-butenoyl moiety (partial structure VII), which was established from ^1^H-^1^H COSY and HMBC data as shown in Figure 4.

The 3-methylbutanoyl moiety (partial structure VIII) in streptcytosine E (**5**) was also assigned from ^1^H-^1^H COSY and HMBC correlations (Figure 4).

**Table 2:** ^13^C (100 MHz) and ^1^H (400 MHz) NMR data for streptcytosines B–E (**2**–**5**) (CD_3_OD).

	2		3		4		5	
C#	δ_C_	δ_H_*,* mult. (*J* in Hz)	δ_C_	δ_H_*,* mult. (*J* in Hz)	δ_C_	δ_H_*,* mult. (*J* in Hz)	δ_C_	δ_H_*,* mult. (*J* in Hz)
2	156.6		155.5		154.2		157.8	
4	164.6		164.2		164.3		164.8	
5	98.4	7.34, d (7.5)	98.3	7.30, d (7.6)	98.2	7.26, d (7.5)	99.2	7.47, br d (7.6)
6	146.8	8.19, d (7.5)	147.5	8.23, d (7.6)	147.0	8.21, d (7.5)	146.7	8.12, d (7.6)
8	166.2		171.3		168.2		176.5	
9	115.7	6.07, d (14.6)	133.5		118.7	5.95, qq (1.3, 1.3)	47.3	2.32, d (7.2)
10	150.7	8.01, d (14.6)	137.4	6.71, q (7.0)	161.3		27.1	2.10, m
11	14.7	2.42, s	14.7	1.89, d (7.0)	28.0	1.99, d (1.2)	22.7	0.99, d (6.7)
12	––	––	12.2	1.90, s	20.8	2.24, d (1.2)	22.7	0.99, d (6.7)
1′	85.0	5.71, dd (9.9, 2.1)	85.0	5.71, dd (10.1, 2.2)	84.9	5.71, dd (10.0, 2.2)	84.9	5.70, dd (9.9, 1.8)
2′	32.4	(a) 2.15, m (b) 1.67, m	32.4	(a) 2.16, m (b) 1.67, m	32.4	(a) 2.15, m (b) 1.67, m	32.4	(a) 2.15, m (b) 1.66, m
3′	31.7	(a) 1.67, m (b) 2.13, m	31.7	(a) 1.67, m (b) 2.13, m	31.6	(a) 1.67, m (b) 2.12, m	31.7	(a) 1.66, m (b) 2.12, m
4′	71.8	3.28, m	71.7	3.28, m	71.8	3.27, m	71.8	3.28, m
5′	80.7	3.51, dq (9.1, 6.1)	80.7	3.51, dq (9.2, 6.2)	80.6	3.51, dq (9.1. 6.1)	80.6	3.50, dq (9.2, 6.2)
6′	18.6	1.34, d (6.1)	18.6	1.34, d (6.2)	18.6	1.33, d (6.1)	18.6	1.33, d (6.2)

### 2.3. Anti-Mycobacterial Activity of Compounds **1**–**11**

The antibacterial activities of compounds **1**–**11 ** against *M. smegmatis* NBRC 3207 were evaluated using the paper disc method [19], and MICs were determined by the liquid microdilution method using 96-well plastic plates (Table 3).

Compounds **1**, **7**–**9**, and **11** showed activity against *M. smegmatis* at 5 µg/disc. Amicetin (**9**) was reported to inhibit the growth of *M. tuberculosis* [20] and showed strong activity against *M. smegmatis* in our experiment. The inhibition activity of compounds **1** and **7** (MIC = 32 µg/mL) was about a half of that of compounds **8** (MIC = 16 µg/mL). Therefore, the 2-methylserine moiety attached to the PABA unit will be important for the anti-mycobacterial activities of these compounds.

On the other hand, compounds **2**–**6** were not active against *M. smegmatis* at 50 µg/disc. Consequently, the amino sugar (amosamine) and/or PABA moieties will be essential for anti-mycobacterial activity.

**Table 3:** Anti-mycobacterial activities (inhibition zone: mm) of compounds **1**–**11** against *Mycobacterium smegmatis* NBRC 3207.

Compound	5 μg/disc	10 μg/disc	MIC (μg/mL)
**1**	9	12	32
**2**	–– ^a^	––	n.d.^b^
**3**	––	––	n.d.
**4**	––	––	n.d.
**5**	––	––	n.d.
**6**	––	––	n.d.
**7**	9	13	32
**8**	18	24	16
**9**	21	26	8
**10**	––	12	>64
**11**	9	12	64
streptomycin sulfate	30	38	0.50

^a^: An inhibition zone was not detected. ^b^: Not determined.

## 3. Experimental Section

### 3.1. General Experimental Procedures

Optical rotations were measured with a JASCO P-2300 digital polarimeter (JASCO, Ltd., Tokyo, Japan). UV spectra were obtained on a Hitachi U-3310 UV-Visible spectrophotometer (Hitachi, Ltd., Tokyo, Japan) and IR spectra on a PerkinElmer Spectrum One Fourier transform infrared spectrometer (Waltham, MA, USA). NMR spectral data were obtained by a JEOL JNM-AL-400 NMR spectrometer (JEOL Ltd., Tokyo, Japan; 400 MHz for ^1^H and 100 MHz for ^13^C) in CD_3_OD (δ_H_ 3.31, δ_C _49.0). High-resolution FAB mass spectra were recorded on a JEOL JMS-MS 700 mass spectrometer (JEOL Ltd., Tokyo, Japan). Preparative HPLC was conducted using a Toyosoda CCPU instrument with a Tosoh UV-8010 detector.

### 3.2. Isolation, Identification, and Fermentation of Producing Bacterium

The strain TPU1236A was isolated from a seawater sample collected at Iriomote Island in Okinawa, Japan in September, 2012. Approximately 1 mL of seawater was mixed with 25 mL of sterilized seawater with 1.0% SDS, and 50 μL of the mixture were developed on an agar plate (glycerol 0.6%, arginine 0.1%, K_2_HPO_4_ 0.1%, MgSO_4_ 0.05%, agar 1.5%, and antibiotics (cycloheximide 100 μg/mL and rifampin 5 μg/mL) in natural seawater) [21]. Identification of the strain TPU1236A was carried out by comparing the partial sequence of the 16S rRNA gene. *S. badius* (732/732, 100%) and *S. sindenensis* (730/730, 100%) showed the highest similarity. Therefore, this producing strain TPU1236A was identified as *Streptomyces* sp.

The culture of strain TPU1236A was maintained on a Waksman agar slant (glucose 1%, peptone 0.5%, meat extract 0.5%, NaCl 0.3%, and agar 1.2% in deionized water) at 4 °C. The mycelia grown on the slant culture were inoculated in a 100-mL Erlenmeyer flask containing 40 mL of a seed medium (ASW-A medium: soluble starch 2%, glucose 1%, peptone 0.5%, yeast extract 0.5%, and CaCO_3_ 0.3% in natural seawater, pH 7.0), and the flasks were shaken (150 rpm) at 25 °C for 3 days. The seed culture (2 mL) was transferred into 500-mL Erlenmeyer flasks, each containing 200 mL of the same medium, and incubated at 25 °C for 7 days at 150 rpm.

### 3.3. Extraction and Isolation of Compounds **1**–**11**

The whole broth (10.0 L) was filtered and the mycelia were extracted with MeOH (1 L). The broth filtrate was adsorbed on Diaion HP-20 (500 mL) as a column, and the resin was washed with water (2 L) and eluted with MeOH (2 L). The eluate from HP-20 was combined with the mycelial extract to give about 1.5 g of a solid material, which was separated on an ODS silica gel column by stepwise elution with MeOH in H_2_O to obtain 11 fractions. Collismycin B (**10**, 11.2 mg) was obtained as white crystals from the 70% MeOH fraction. The 70% MeOH fraction was purified by preparative HPLC (a PEGASIL ODS SP100 column, 250 mm × 10 mm, eluted with MeOH–H_2_O = 6:4 containing 0.05% TFA, 2.0 mL/min, detected at UV 210 nm), and streptcytosine A (**1**, 12.3 mg), plicacetin (**7**, 21.3 mg), and SF2738 C (**11**, 6.8 mg) were eluted at 9.9, 15.0, and 12.2 min, respectively. The 60% MeOH fraction was subjected to HPLC (ODS) with 45% MeOH in H_2_O containing 0.05% TFA at 2 mL/min to afford streptcytosines B (**2**, 3.2 mg, 16.8 min), C (**3**, 7.2 mg, 17.7 min), D (**4**, 8.1 mg, 19.3 min), and E (**5**, 5.1 mg, 20.2 min), de-amosaminyl-cytosamine (**6**, 12.0 mg, 20.1 min), and bamicetin (**8**, 20.5 mg, 5.8 min). Amicetin (**9**, 25.0 mg, 8.5 min) was isolated by HPLC with 60% MeOH in H_2_O (0.05% TFA) from the 80% MeOH fraction.

**Streptcytosine A (1)**: obtained as a colorless oil; [α]^23^_D_ +72 (c 0.1, MeOH); UV λ_max_ (0.1 M HCl) nm (log ε): 269 (4.24), 276 (4.24), 317 (4.28); IR ν_max_ (KBr) cm^–1^: 3500–3400, 2920, 1712, 1677, 1639, 1613, 1385, 1262, 1093, 802; HRFABMS (*m/z*) found: 631.3110, calcd: 631.3092 [M + H]^+^ for C_30_H_43_N_6_O_9_,; ^1^H and ^13^C NMR data, see Table 1.

**Streptcytosine B (2)**: obtained as a pale yellow oil; [α]^23^_D_ +24 (c 0.1, MeOH); UV λ_max_ (0.1 M HCl) nm (log ε): 249 (3.94), 338 (4.09); IR ν_max_ (KBr) cm^–1^: 3500–3400, 2927, 1683, 1646, 1616, 1576, 1493, 1385, 1247, 1092; HRFABMS (*m/z*) found: 326.1180, calcd: 326.1175 [M + H]^+^ for C_14_H_20_N_3_O_4_S; ^1^H and ^13^C NMR data, see Table 2.

**Streptcytosine C (3)**: obtained as a pale yellow oil; [α]^23^_D_ +36 (c 0.1, MeOH); UV λ_max_ (0.1 M HCl) nm (log ε): 257 (3.95), 311 (4.18); IR ν_max_ (KBr) cm^–1^: 3500–3400, 2928, 1683, 1646, 1613, 1561, 1489, 1385, 1262, 1092; HRFABMS (*m/z*) found: 308.1619, calcd: 308.1610 [M + H]^+^ for C_15_H_22_N_3_O_4_; ^1^H and ^13^C NMR data, see Table 2.

**Streptcytosine (4)**: obtained as a pale yellow oil; [α]^23^_D_ +68 (c 0.1, MeOH); UV λ_max_ (0.1 M HCl) nm (log ε): 217 (3.96), 263 (4.16), 309 (4.32); IR ν_max_ (KBr) cm^–1^: 3500–3400, 2937, 1735, 1674, 1634, 1561, 1492, 1396, 1272, 1095; HRFABMS (*m/z*) found: 308.1601, calcd: 308.1610 [M + H]^+^ for C_15_H_22_N_3_O_4_; ^1^H and ^13^C NMR data, see Table 2.

**Streptcytosine (5)**: obtained as a pale yellow oil; [α]^23^_D_ +62 (c 0.1, MeOH); UV λ_max_ (0.1 M HCl) nm (log ε): 237 (3.61), 310 (4.04); IR ν_max_ (KBr) cm^–1^: 3500–3400, 2963, 1720, 1677, 1624, 1571, 1494, 1383, 1276, 1095; HRFABMS (*m/z*) found: 310.1763, calcd: 310.1767 [M + H]^+^ for C_15_H_24_N_3_O_4_; ^1^H and ^13^C NMR data, see Table 2.

### 3.4. Anti-Mycobacterial Assay

The anti-mycobacterial assay was carried out using M. smegmatis NBRC 3207 with the paper disc method and liquid microdilution method. The strain NBRC 3207 was obtained from the Biological Resource Center (NBRC), NITE (Chiba, Japan) and maintained in 20% glycerol at −80 °C.

The test microorganism was cultured in Middlebook 7H9 broth (BD, Franklin Lakes, NJ, USA) containing 0.05% polysorbate 80 (BD), 0.5% glycerol, and 10% Middlebook OADC (BD) at 37 °C for 2 days and adjusted to 1.0 × 10^6^ CFU/mL. The inoculum was spread on the above medium containing 1.5% agar in a square plate. Each sample in MeOH was adsorbed to a sterile filter disc (6 mm, Advantec, Tokyo, Japan), and, after evaporation of MeOH, the disc was placed in a plate and incubated for 2 days at 37 °C. Streptomycin sulfate (5 and 10 µg/disc) and MeOH were used as positive and negative controls, respectively. 

MICs were measured by the liquid microdilution method [22]. After 85 μL of Middlebrook 7H9 broth containing 0.05% polysorbate 80, 0.5% glycerol, and 10% Middlebook OADC was added to each well of a 96-well microplate (Corning Inc., Corning, NY, USA), test compound dissolved in MeOH (5.0 μL) was added to each well at the final concentration of 0.125 to 64 μg/mL, respectively. Finally, the test microorganism (10 μL) was added at a concentration of 1.0 × 10^6^ CFU/mL. Microplates were incubated at 37 °C for 2 days. MIC was defined as the lowest concentration of test compound where the test microorganism cannot grow.

## 4. Conclusions

Five new pyrimidine nucleoside antibiotics, named streptcytosines A–E (**1**–**5**), and six known compounds **6**–**11** were obtained from a marine-derived actinomycete, *Streptomyces* sp. TPU1236A, isolated from seawater collected at Iriomote Island in Okinawa, Japan. The anti-mycobacterial activity of the extract of the culture broth was reproduced by new compound **1** (streptcytosine A), plicacetin (**7**), bamicetin (**8**), amicetin (**9**), and SF2738 C (**11**). Compounds **8** and **9** were about two-fold more active than the other three compounds.
